# Hydrodissection performed safely with an injection catheter during robot-assisted radical prostatectomy

**DOI:** 10.1080/2090598X.2022.2146222

**Published:** 2022-11-14

**Authors:** Jotaro Mikami, Jun Ito, Yuki Kohada, Nao Iwamoto, Hiroki Kusumoto, Takashi Kukimoto, Masaaki Oikawa, Yasuhiro Kaiho

**Affiliations:** Division of Urology, Faculty of Medicine, Tohoku Medical and Pharmaceutical University, Sendai, Japan

**Keywords:** Prostatectomy, surgical procedures, recovery of function, nerve, erectile dysfunction

## Abstract

To facilitate nerve preservation during robot-assisted radical prostatectomy (RP), hydrodissection (HD) using an injection catheter was performed. HD during RP is a nerve-sparing technique in which an epinephrine solution is injected into the lateral prostatic fascia to separate it from the prostatic capsule. Although the beneficial effects of HD on postoperative sexual function have been reported, HD has rarely been used in robot-assisted RP. The primary reason may be the potential benefits of robotic surgery, such as less bleeding, magnified surgical view, and fine movement of instruments; another possible reason is the difficulty of handling sharp needles in a narrow intra-abdominal surgical space of robot-assisted RP. For safe fluid injection, we performed HD using an injection catheter – commonly used for endoscopic upper gastrointestinal hemostasis – during robot-assisted RP. The required time to accomplish HD and the safety of the procedure were examined in 15 HD of 11 patients. Approximately 2 minutes (median, 118 seconds; interquartile range, 106–174 seconds) were needed for HD using the injection catheter. All patients had no complications, such as injuries to the intestine, vessels, or other organs. Postoperative bleeding did not occur in any patients. HD with an injection catheter enables surgeons to perform simple and safe nerve preservation during robot-assisted RP.

Nerve-sparing (NS) procedures during radical prostatectomy (RP) are generally performed to enhance postoperative erectile function recovery [1]. However, we sometimes encounter patients in whom performing nerve preservation with the appropriate surgical plane of purpose, such as in intra-fascial or inter-fascial dissections, is difficult. Hydrodissection (HD), in which a solution of epinephrine is injected into the lateral prostatic fascia to separate it from the prostatic capsule, is a technique that was introduced to facilitate nerve preservation [2–4]. Patel et al. reported that HD decreased intraoperative bleeding and improved postoperative sexual function without increasing positive margin status during RP [2]. Although HD is a beneficial technique for exposing the appropriate surgical planes during open RP, it is infrequently used in the era of robot-assisted RP. The primary reason for this is the potential benefits of robotic surgery, such as less bleeding, magnified surgical view, and fine movement of instruments. However, another possible reason is the burden of handling sharp needles in a narrow intra-abdominal surgical space in robot-assisted RP. To perform simple and safe HD during robot-assisted RP, we used an injection catheter commonly used for endoscopic upper gastrointestinal hemostasis. The needle tip can be retracted until just before the puncture, thus preventing accidental puncture of the surrounding organs and tissues.

HD during robot-assisted RP was performed with the approval of the Clinical Research Ethics Board of Tohoku Medical and Pharmaceutical Hospital (approval number: 2021-4-022). An injection catheter with a 22 G needle and a 4 mm long needle tip (Olympus, Tokyo, Japan), commonly used for endoscopic upper gastrointestinal hemostasis, was inserted through a 5-mm assistant port. The operator controlled the catheter using robotic arms and placed the needle tip in the lateral pelvic fascia of the NS side of the prostate. An assistant operator injected approximately 10 mL of saline (Figure 1, Supplemental video 1). An epinephrine solution has been commonly used for HD to prevent bleeding during RP; however, in robot-assisted RP, pure saline can be used for HD without problems. After HD, lateral pelvic fasciae incisions that were hydrodissected by injected saline were placed at the 2 (10) o’clock position of the prostate in the axial section to preserve nerve fibers in the anterolateral and posterolateral aspects of the prostate. Injected saline usually spreads within the lateral pelvic fascia (interfascial dissection); however, it seldom spreads between the lateral pelvic fascia and the prostatic capsule (intra-fascial dissection). If intra-fascial NS is required, the remaining lateral pelvic fasciae, after incising the hydrodissected lateral pelvic fasciae, are incised further until the surface of the prostate is exposed.

**Figure 1.**
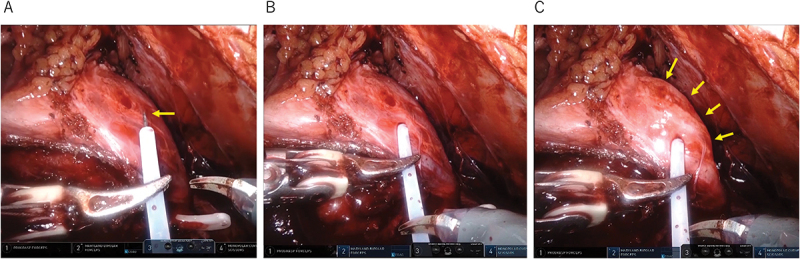
Hydrodissection using an injection catheter. A: The needle tip (yellow arrow) is pushed out immediately before the puncture, which prevents accidental puncture of the surrounding organs and tissues. B: The needle tip is inserted into the lateral pelvic fascia of the nerve-sparing side of the prostate. C: The lateral pelvic fasciae are hydrodissected using injected saline (yellow arrows).

Fifteen HD (eight right sided and seven left sided) were performed on 11 patients during NS robot-assisted RP by three surgeons from December 2019 to September 2020. The clinical characteristics and operative parameters were examined from clinical records. The duration of HD, defined as the time from insertion of the catheter into the body by the assistant operator to catheter removal after saline injection, was measured. Resection margin status on HD sides was assessed. In addition, any complications during HD, such as injury to the intestine, vessels, or other organs, and postoperative bleeding were examined retrospectively by reviewing the surgical movies and clinical records.

The clinical characteristics and operative parameters are shown in Table 1. Approximately 2 minutes (median, 118 seconds; interquartile range, 106–174 seconds) were required from insertion to removal of the catheter after saline injection. Insertion and handling of the catheter were performed safely in all patients without any complications. Postoperative bleeding did not occur in any of the patients. One patient (9%) had positive surgical margin at final pathology, however, PSA level has been under 0.01 ng/mL for 18 months after surgery.

**Table 1.** Clinical characteristics and operative parameters.

		HD (n = 11)
Parameter		Median (IQR) or Number (%)
Age (year)		65 (64.5–69.5)
Major Comorbidities	Hypertension	2 (18%)
	Diabetes mellitus	1 (9%)
Preoperative PSA (ng/ml)		6.2 (5.7–9.5)
No. biopsy Gleason score	3 + 3	2 (18%)
	3 + 4	6 (55%)
	4 + 3	3 (27%)
Preoperative EPIC score	Urinary function score	92.6 (84.1–93.4)
	Urinary bother score	100 (97.2–100)
	Sexual function score	13.9 (5.5–16.2)
	Sexual bother score	100 (75–100)
Operative parameters,	Operative time (min)	256 (232–282)
	Blood loss (ml)	175 (60–287)
	Duration of HD (sec)	118 (106–174)
Excised prostate weight (g)		41 (36–58)
Final pathologic stage	pT2b	9 (82%)
	pT2c	1 (9%)
	pT3	1 (9%)
Positive surgical margin on HD side		1 (9%)

HD: hydrodissection, IQR: interquartile range, PSA: prostate specific antigen, EPIC: Expanded Prostate Cancer Index Composite,

HD with an injection catheter enables surgeons to perform simple and safe nerve preservation during robot-assisted RP. Future studies are needed to confirm the efficacy and clinical applicability of HD with an injection catheter.

## Supplementary Material

Supplemental Material

## Abbreviations list

HD: hydrodissection

NS: nerve sparing

RP: radical prostatectomy
